# Observed High Coinfection Rates Seem To Be a Result of Overlapping Plaques

**DOI:** 10.1128/mBio.01000-17

**Published:** 2017-08-08

**Authors:** Nir Drayman

**Affiliations:** Institute for Molecular Engineering and Institute for Genomics and Systems Biology, University of Chicago, Chicago, Illinois, USA; Vanderbilt University Medical Center

**Keywords:** methodology, plaque assay, poliovirus

## LETTER

In their recent paper, Aguilera et al. (1) investigated the rate of coinfection by poliovirus. It seems that the results that they obtained using two different methods are at odds. When infecting cells at a very low multiplicity of infection (MOI) (10^−5^ PFU/cell) and assaying viral progeny production using plaque assays, the authors found that 5 to 7% of plaques originated from more than a single virus, far above the rate expected from a random distribution of viruses according to Poisson’s law (which is ~10^−10^ for the used MOI). However, when the authors infected cells at a somewhat higher MOI (10^−2^ PFU/cell) and assayed viral gene expression at the single-cell level using flow cytometry, the percentage of cells infected by more than one virus was as expected from Poisson's law (0.0048% observed versus 0.005% expected).

How can these seemingly contradicting results be reconciled? At least two possibilities come to mind: (i) due to the spatial constraints of the plaque assay, some plaques (each initiated from a single virus) overlap, resulting in the appearance of coinfection, and (ii) cells infected by more than one virus have a higher probability of successfully forming a plaque.

**FIG 1:**
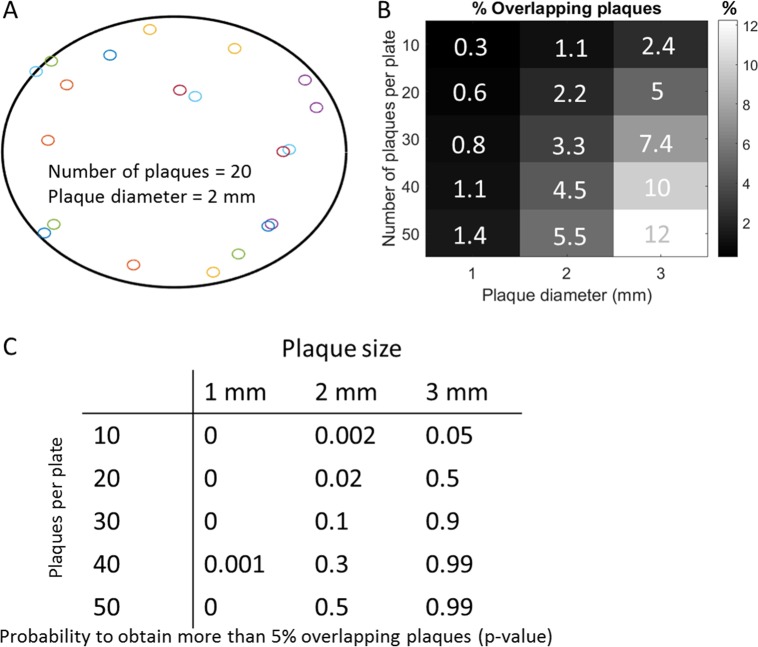
Simulations of plaque assays show high prevalence of overlapping plaques. (A) Example of a virtual plaque assay. Each circle is a single plaque. This example shows 20 plaques, each with a 2-mm diameter, four of which overlap. (B) Percentages of overlapping plaques in 1,000 simulations for each combination of plaque size and number of plaques per plate. (C) *P* values calculated for observing >5% coinfection rates when “picking” 120 plaques under each condition.

While the second option is intriguing, the first seems more likely and is supported by the simulations presented below. Using 10-cm plates, the authors picked ~120 plaques from plates harboring between a few and 50 plaques, with an average of 20 plaques per plate. The average size of poliovirus plaques at 48 h is reported to be between 1 and 3 mm (2). Using these parameters, simulations can be performed where a virtual 10-cm plate is randomly seeded with plaques and the percentage of overlapping plaques calculated (Fig. 1A). Repeating this 1,000 times for each combination of plaque size and number of plaques per plate results in a good estimate of the percentage of overlapping plaques (Fig. 1B). By virtually “picking” ~120 plaques in each simulation run, *P* values can also be calculated by counting the number of simulations that resulted in >5% overlapping plaques (Fig. 1C).

These simulations suggest that due to the spatial constraints of the plaque assay, one can expect anywhere between 0.3 and 12% of plaques to overlap to some degree (depending on the plaque size and the number of plaques in the plate), resulting in seemingly high coinfection rates. This suggests that the discrepancy between the estimates of coinfection rates by the plaque assay and flow cytometry are most likely due to the technical limitation of resolving overlapping plaques.
